# The rice RNase P protein subunit Rpp30 confers broad‐spectrum resistance to fungal and bacterial pathogens

**DOI:** 10.1111/pbi.13612

**Published:** 2021-05-17

**Authors:** Wei Li, Yehui Xiong, Lien B. Lai, Kai Zhang, Zhiqiang Li, Houxiang Kang, Liangying Dai, Venkat Gopalan, Guo‐Liang Wang, Wende Liu

**Affiliations:** ^1^ State Key Laboratory for Biology of Plant Diseases and Insect Pests Institute of Plant Protection Chinese Academy of Agricultural Sciences Beijing China; ^2^ Hunan Provincial Key Laboratory for Biology and Control of Plant Diseases and Insect Pests and College of Plant Protection Hunan Agricultural University Changsha Hunan China; ^3^ Department of Plant Pathology The Ohio State University Columbus OH USA; ^4^ Department of Chemistry and Biochemistry Center for RNA Biology The Ohio State University Columbus Ohio USA

**Keywords:** OsRpp30, HDT701, RNase P, *Pyricularia oryzae* (syn. *Magnaporthe oryzae*), *Xanthomonas oryzae* pv. *oryzae*

## Abstract

RNase P functions either as a catalytic ribonucleoprotein (RNP) or as an RNA‐free polypeptide to catalyse RNA processing, primarily tRNA 5′ maturation. To the growing evidence of non‐canonical roles for RNase P RNP subunits including regulation of chromatin structure and function, we add here a role for the rice RNase P Rpp30 in innate immunity. This protein (encoded by *LOC_Os11g01074)* was uncovered as the top hit in yeast two‐hybrid assays performed with the rice histone deacetylase HDT701 as bait. We showed that HDT701 and OsRpp30 are localized to the rice nucleus, *OsRpp30* expression increased post‐infection by *Pyricularia oryzae* (syn. *Magnaporthe oryzae*), and OsRpp30 deacetylation coincided with HDT701 overexpression *in vivo*. Overexpression of *OsRpp30* in transgenic rice increased expression of defence genes and generation of reactive oxygen species after pathogen‐associated molecular pattern elicitor treatment, outcomes that culminated in resistance to a fungal (*P*. *oryzae*) and a bacterial (*Xanthomonas oryzae* pv. *oryzae*) pathogen. Knockout of *OsRpp30* yielded the opposite phenotypes. Moreover, HA‐tagged OsRpp30 co‐purified with RNase P pre‐tRNA cleavage activity. Interestingly, OsRpp30 is conserved in grass crops, including a near‐identical C‐terminal tail that is essential for HDT701 binding and defence regulation. Overall, our results suggest that OsRpp30 plays an important role in rice immune response to pathogens and provides a new approach to generate broad‐spectrum disease‐resistant rice cultivars.

## Introduction

Acetylation and deacetylation of histones in eukaryotes are two dynamic and reversible post‐translational modifications (PTMs) which are regulated by the antagonistic enzymes histone acetyltransferases (HATs) and histone deacetylases (HDACs), respectively (Fierz and Poirier, 2019; Shahbazian and Grunstein, 2007). In plants, these enzymes are known to play a crucial role in many cellular processes including plant growth, development, hormone signalling, responses to abiotic stresses and immunity to pathogens (Ma *et al.,* 2013). HATs and HDACs function in plant innate immunity by controlling the expression of defence genes (Ding and Wang, 2015; Song and Walley, 2016).

The rice genome contains eight HATs and 17 HDACs (Ding *et al.,* 2012; Fu *et al.,* 2007; Liu *et al.,* 2012). The HDACs are classified into three families including the plant‐specific HD2, which comprises two rice members HDT701 and HDT702 (Fu *et al.,* 2007; Zhao *et al.,* 2015). We previously demonstrated the function of HDT701 in suppressing innate immunity in rice to pathogens (Ding *et al.,* 2012). Overexpression of *HDT701* in transgenic rice decreased histone H4 acetylation and enhanced susceptibility to *Pyricularia oryzae* (syn. *Magnaporthe oryzae*) and *Xanthomonas oryzae* pv. *oryzae* (*Xoo*). In contrast, silencing of *HDT701* in transgenic rice elevated histone H4 acetylation as well as transcription of pattern recognition receptor (PRR) and defence genes, increased generation of reactive oxygen species (ROS) after pathogen‐associated molecular pattern (PAMP) elicitor treatment and enhanced resistance to both *P*. *oryzae* and *Xoo*. These results support our working model that HDT701 negatively regulates innate immunity by modulating the level of histone H4 acetylation and expression of PRR and defence genes in rice (Ding *et al.,* 2012). HDT701 has also been reported to negatively regulate plant abiotic stress tolerance (e.g. ABA, salt) and to function in seed germination and flowering (Cho *et al.,* 2018; Zhao *et al.,* 2015). Here, our efforts to identify cellular proteins that may mediate the biological functions of rice HDT701 led to the finding of a surprising partner: OsRpp30, a putative subunit of RNase P.

RNase P was initially identified as the ubiquitous endoribonuclease for cleaving 5' end of precursor tRNAs to make mature tRNAs (Altman, 2007; Gopalan *et al.,* 2018; Lai *et al.,* 2010). Although an RNA‐free form of RNase P was discovered later (Daniels *et al.,* 2019; Holzmann *et al.,* 2008), OsRpp30 is a subunit of only the RNP form. The RNase P RNP consists of one catalytic RNA and a varying number of RNase P protein (Rpp) subunits: one in bacteria, up to five in archaea, and 9–10 in eukaryotes (Altman, 2007; Lai *et al.,* 2010; Samanta *et al.,* 2016). In addition to its principal role in tRNA 5' biogenesis, RNase P also cleaves long non‐coding RNAs (ncRNAs), rRNAs and mRNAs (Hernandez‐Cid *et al.,* 2012; Jarrous, 2017; Jarrous and Gopalan, 2010). Moreover, RNase P is required for efficient transcription of various small ncRNAs, including tRNAs, 5S rRNA, SRP RNA and U6 snRNA (Jarrous, 2017; Jarrous and Reiner, 2007). Recent studies show that RNase P is also involved in regulating chromatin structure and function. For instance, the human RNase P subunits Rpp21, Rpp29 and Pop1 act as a repressor of histone H3.3 chromatin assembly (Newhart *et al.,* 2016; Shastrula *et al.,* 2018). Although there have been indications of an RNase P RNP in plants (Franklin *et al.,* 1995; Krehan *et al.,* 2012; Pulukkunat, 2002), a firm knowledge of its subunit composition has proven elusive. Interestingly, mutating *Arabidopsis* Rpp30 (AtRpp30) leads to defective progression of the gametophytic division during female gametogenesis and weakens the plant pollen tube, resulting in reduced transmission through the male gametes (Wang *et al.,* 2012). The mechanistic basis for how AtRpp30 contributes to reproduction and development is unknown.

Here, we found that overexpression of *OsRpp30* in transgenic rice increases defence gene expression and ROS generation after a PAMP elicitor treatment, outcomes that culminated in resistance to bacterial and fungal pathogens. Knockout of *OsRpp30* yielded the opposite phenotypes. In addition, OsRpp30 is deacetylated upon HDT701 overexpression *in vivo* and is associated with partially purified rice RNase P that exhibits tRNA 5′‐processing. The C‐terminal tail of OsRpp30 necessary for binding HDT701 is also found in wild rice species and other cereals and is required for inducing expression of defence genes. Our results uncover a link between plant immunity and a protein known to be essential for tRNA processing.

## Results

### OsRpp30, but not its paralogs, interacts with HDT701 *in vivo*

**Figure 1:**
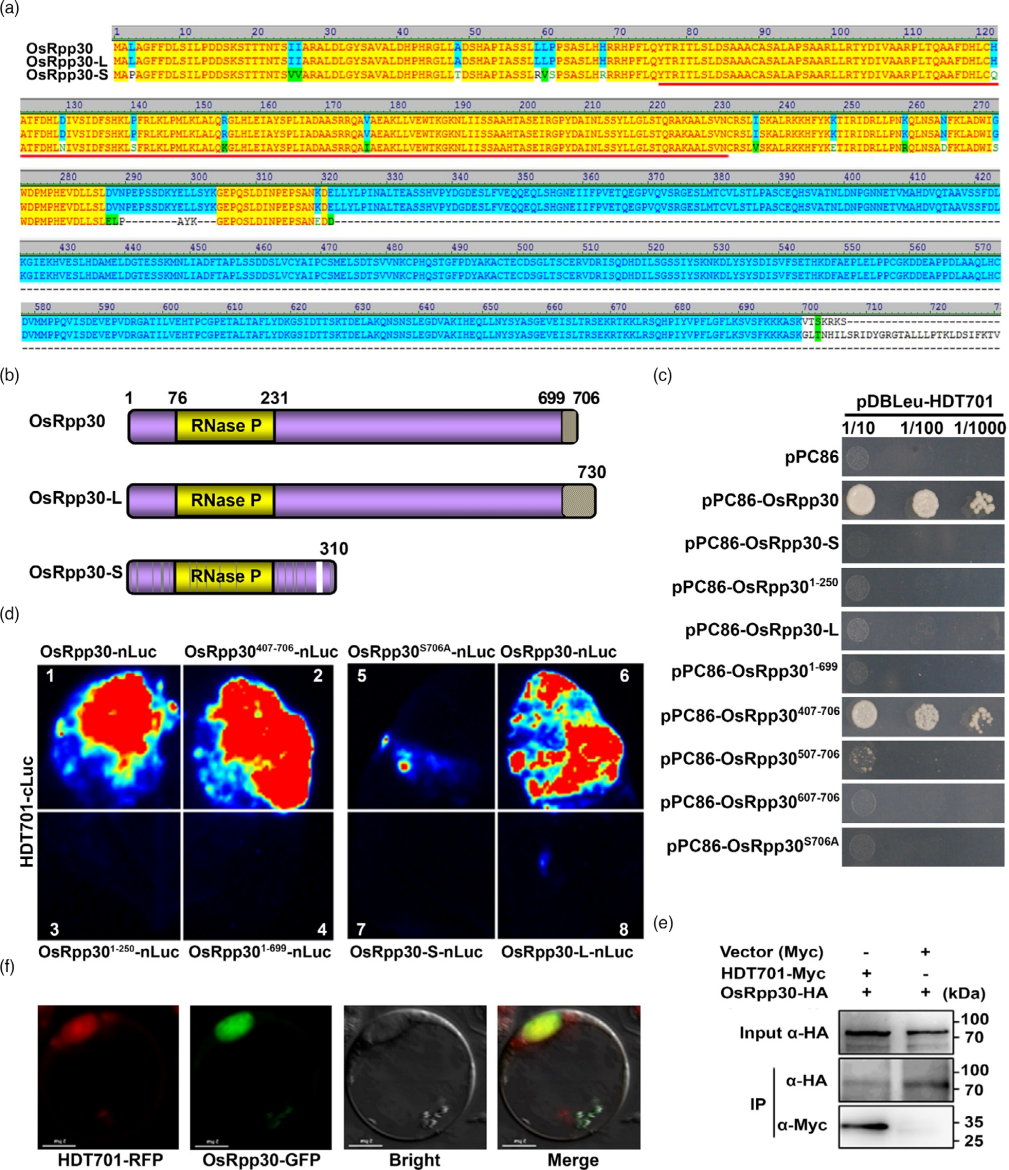
OsRpp30 interacts with HDT701. (a) Alignment of OsRpp30, OsRpp30‐L and OsRpp30‐S. (b) Schematic of the three OsRpp30 proteins outlining regions of homology and divergence. Note that OsRpp30 is identical to OsRpp30‐L except in the tail end of their C‐terminus. The red (a) and yellow (b) bars denote the domain that is present in Rpp30 from other eukaryotes. (c) OsRpp30 interacts with HDT701 but not its paralogs OsRpp30‐S and OsRpp30‐L. Y2H assays were performed using SD‐Leu‐Trp‐His medium containing 20 mm 3‐AT. (d) LCI assay in *Nicotiana* *benthamiana* leaves of the interaction between HDT701 and various derivatives of OsRpp30. Each of the eight quadrants was infiltrated with HDT701‐cLuc and the indicated construct. Note that quadrants 1 and 6 are repeats. (e) Co‐IP analysis of the interaction between OsRpp30 and HDT701 in rice. Protoplasts were transfected with the indicated plasmids, and total protein was used as input for immunoprecipitation with anti‐HA beads before immunoblotting with the indicated antibodies. (f) Co‐localization of OsRpp30 and HDT701 in the rice nucleus. Confocal fluorescence microscopy of rice protoplasts co‐transfected with *OsRpp30‐GFP* and *HDT701‐RFP* plasmids.

To identify HDT701‐interacting proteins in rice, we screened a yeast two‐hybrid (Y2H) rice cDNA library using HDT701 as bait. Sequencing of positive clones showed that a gene (*LOC_Os11g01074*) encoding the putative RNase P protein subunit Rpp30 was the top hit. BLAST searches of the rice genome revealed two paralogs of *OsRpp30* (*LOC_Os12g07680* and *LOC_Os12g01060*) that we named *OsRpp30‐S* (shorter) and *OsRpp30‐L* (longer) based on their lengths relative to *OsRpp30* (Figures 1a,b). While OsRpp30‐S aligns to the N‐terminal half of OsRpp30 with 96% identity, OsRpp30‐L is identical to OsRpp30 up to amino acid (aa) 699, but its last 31 aa have no homology to the last seven aa in OsRpp30 (Figure 1a,b). It is notable that the C‐terminal half of OsRpp30 is a new module specific to plants and missing in other eukaryotes.

Y2H assays revealed that HDT701 interacted only with OsRpp30, not with OsRpp30‐S or OsRpp30‐L (Figure 1c). HDT701 also did not interact with just the first 250 aa (OsRpp30^1‐250^) or 699 aa (OsRpp30^1‐699^) of OsRpp30, regions that are equivalent to OsRpp30‐S or OsRpp30‐L, respectively. While OsRpp30^407‐706^ showed robust interaction, further deletions (OsRpp30^507‐706^ and OsRpp30^607‐706^) abrogated HDT701 binding. These results point to the importance of the C‐terminal half of OsRpp30, particularly its last seven aa that is absent in OsRpp30‐L. Remarkably, mutating just the last aa of OsRpp30 (S706A) eliminated all binding with HDT701.

We employed multiple approaches to confirm the above findings *in planta*. First, we authenticated the HDT701‐OsRpp30 interaction with a luciferase complementation imaging (LCI) assay in *Nicotiana benthamiana* (Figure 1d). Mirroring the Y2H results, strong luminescence was detected when HDT701‐cLuc and OsRpp30‐nLuc were co‐expressed under the control of the cauliflower mosaic virus 35S promoter in *N*. *benthamiana* leaves. Such luminescence was greatly diminished when (i) OsRpp30‐S or OsRpp30‐L replaced OsRpp30, (ii) the last seven aa of OsRpp30 was omitted, or (iii) the mutation S706A was introduced in OsRpp30. Second, following co‐expression of *OsRpp30‐HA* and *HDT701‐Myc* driven by the 35S promoter in rice protoplasts, we performed a co‐immunoprecipitation (co‐IP) test. The HA antibody successfully pulled down both OsRpp30‐HA and HDT701‐Myc, indicating their interaction in rice (Figure 1e). Finally, because HDT701 functions in the rice nucleus (Ding *et al.,* 2012), we postulated likewise for OsRpp30. To test this idea, we first co‐transfected rice protoplasts with *OsRpp30‐green fluorescent protein* (*GFP*) and *HDT701‐red fluorescent protein* (*RFP*) fusion constructs, both under the control of the 35S promoter. Confocal fluorescence microscopy analysis then revealed that both OsRpp30‐GFP and HDT701‐RFP indeed co‐localized in the nucleus (Figure 1f). Together, the above results unambiguously demonstrated that HDT701 and OsRpp30 interact and are present in the same cellular compartment.

### OsRpp30 positively regulates rice immunity

**Figure 2:**
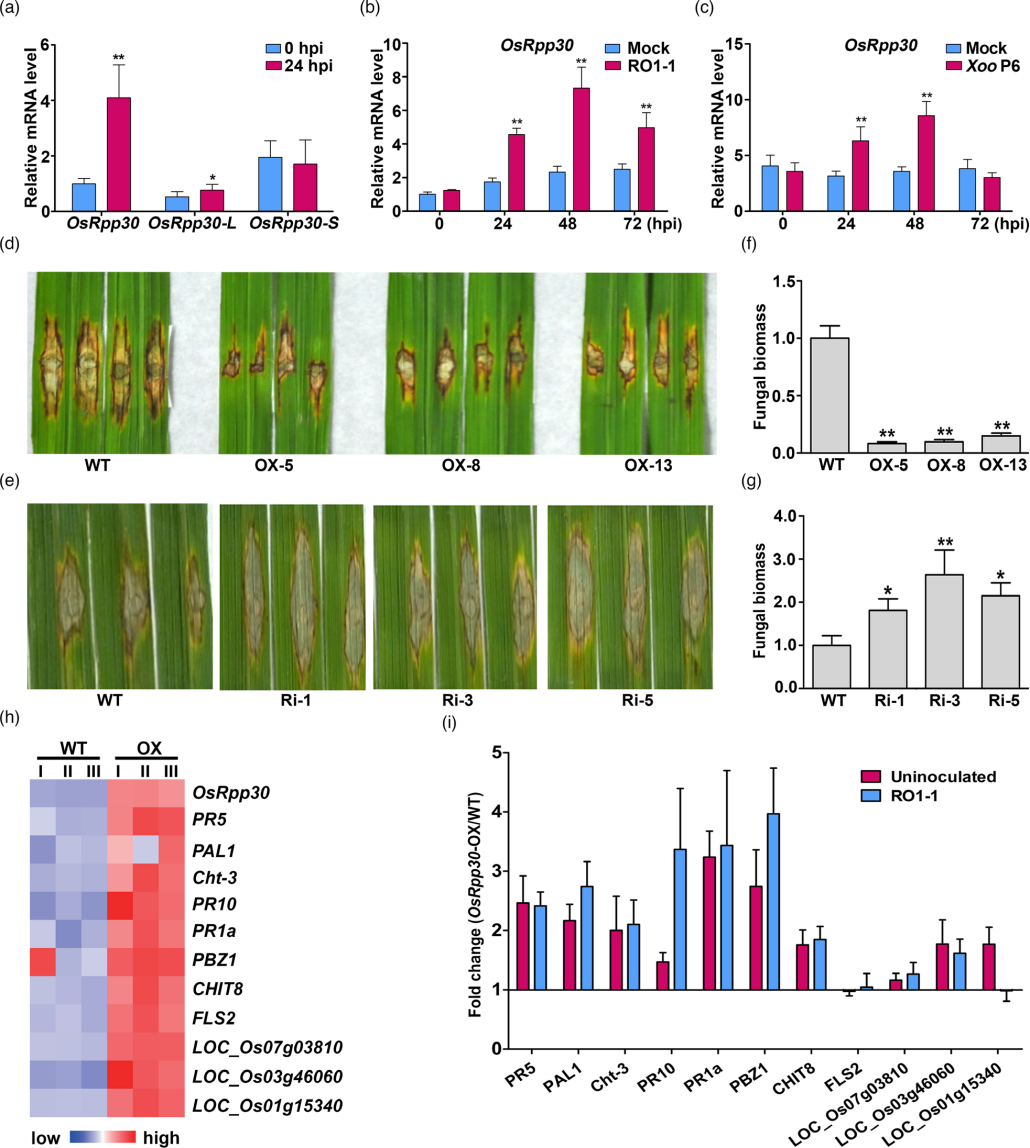
OsRpp30 positively regulates plant disease resistance. (a) Expression of *OsRpp30, OsRpp30‐L* and *OsRpp30‐S* at 0 and 24 h post‐inoculation (hpi) with *Pyricularia* *oryzae* RO1‐1. (b) Expression of *OsRpp30* induced by *P. oryzae* RO1‐1 infection by a spraying inoculation method. (c) Expression of *OsRpp30* induced by *Xoo* P6 infection by a leaf‐clipping inoculation method. (d) Lesion on WT and *OsRpp30*‐OX (OX) rice leaves at 13 dpi with *P. oryzae* RO1‐1. (e) Lesion on WT and *OsRpp30*‐RNAi (Ri) rice leaves at 13 dpi with *P*. *oryzae* RO1‐1. (f) Relative fungal biomass in the necrotic regions of the leaves in panel d. Fungal growth was assessed by qPCR of the fungal *Pot2* gene and normalized to rice *Ubiquitin* gene. (g) Relative fungal biomass in the necrotic regions of the leaves in panel e. Fungal growth was assessed as described in panel f. (a–c, f–g) These data are the mean of at least three biological replicates ± SE. Asterisks denote significant difference based on nested ANOVA (*P < 0.05, **P < 0.01). (h) Heatmap showing the differentially expressed genes (DEGs) associated with immune responses and development in *OsRpp30*‐OX plants and WT plants without inoculation. (i) Expression fold change assessed by RT‐qPCR of the genes (in panel h) in uninoculated and inoculated (at 24 hpi with *P*. *oryzae* RO1‐1) *OsRpp30*‐OX relative to WT plants. A fold change of 1.0 signifies no difference between *OsRpp30*‐OX and WT plants. The data reported are the mean of three replicates of RT‐qPCR ± SE.

To determine whether OsRpp30 is involved in rice immunity akin to HDT701 (Ding *et al.,* 2012), we assessed *OsRpp30* mRNA levels by quantitative RT‐PCR (RT‐qPCR) after spray inoculation of wild‐type (WT) plants with the compatible fungal pathogen *P*. *oryzae* RO1‐1. Indeed, *OsRpp30* mRNA level increased fourfold at 24 h post‐inoculation (h.p.i), while a modest (˜1.5‐fold) increase was observed for *OsRpp30‐L* and none for *OsRpp30‐S* (Figure 2a). In a post‐inoculation time course analysis, *OsRpp30* mRNA level peaked at 48 hpi and remained elevated even at 72 hpi (Figure 2b). To explore the broader significance with a second pathogen, we determined *OsRpp30* transcript levels in WT rice plants inoculated with the bacterial blight pathogen *Xoo* Race 6 POX99 (P6). Likewise, *OsRpp30* expression increased and peaked at 48 hpi (Figure 2c). Together with the finding of HDT701–OsRpp30 interaction, these results suggest that *OsRpp30*, but not *OsRpp30‐L* or *OsRpp30‐S*, is involved in rice immunity.

We next examined how overexpression or knockdown of *OsRpp30* affect rice immunity. We generated transgenic plants in which *HA‐OsRpp30* was overexpressed (OX, four‐ to sevenfold) from a maize ubiquitin promoter (Figure S1a). To evaluate the resistance phenotype of *OsRpp30*‐OX compared to WT plants, we punch‐inoculated these plants with the compatible *P*. *oryzae* RO1‐1. Consistent with the enhanced expression of *OsRpp30* post‐inoculation playing a role in rice immunity, *OsRpp30*‐OX plants displayed significantly smaller lesions compared to the WT post‐inoculation (Figure 2d). Interestingly, while *OsRpp30*‐OX plants showed no developmental or growth defects, their seedlings displayed spontaneous cell death under high‐humidity growth conditions such as in sealed tissue culture containers, likely on account of stress (Figure S1b). To corroborate findings from the overexpression lines, we also generated transgenic plants in which RNAi was used to knock down *OsRpp30* (Figures S1c,d). In direct contrast to *OsRpp30*‐OX plants, *OsRpp30*‐RNAi plants showed larger lesions upon punch inoculation with *P*. *oryzae* RO1‐1 relative to WT plants (Figure 2e). Correspondingly, the fungal biomass of *P*. *oryzae* RO1‐1 was much lower post‐inoculation in *OsRpp30*‐OX plants and higher in *OsRpp30*‐RNAi plants compared to the WT (Figure 2f,g). To determine the reciprocal regulation of *OsRpp30* and *HDT701* during rice immunity, we determined *HDT701* mRNA levels in *OsRpp30*‐RNAi and *OsRpp30*‐OX plants. We found that *HDT701* mRNA levels did not change by more than 25% upon knockdown or overexpression of *OsRpp30* (Figure S1e). Likewise, *OsRpp30* mRNA levels were only somewhat affected (<25%) by knockout and overexpression of *HDT701* (Figure S1f; see below for the description of the HDT701 knockout lines).

To elucidate the *OsRpp30‐*mediated resistance mechanism(s), we examined gene expression differences between *OsRpp30*‐OX and WT plants without any inoculation using RNA sequencing (RNA‐seq). Bioinformatic analyses identified 247 differentially expressed genes (DEGs) with at least twofold change: 172 were up‐ and 75 were down‐regulated in the *OsRpp30*‐OX plants (Table S1). Of these, 120 up‐regulate d and 46 down‐regulate d DEGs are annotated with different gene ontology (GO) terms; the rest are unannotated. Eight of the 120 up‐regulate d (˜7%) and five of the 46 down‐regulate d (˜11%) DEGs are annotated as ‘defence response’. Compared to the 445 ‘defence response’ genes of the 22 600 annotated rice genes in total (˜2%), our data sets showed that overexpression of *OsRpp30* clearly affect expression of defence genes more selectively than by chance [7% (up) and 11% (down) vs. 2%]. Consistent with a normal growth and lack of any gross morphological difference in *OsRpp30*‐OX compared to WT plants, we found only a few development‐related genes among the DEGs (e.g. flowering promoting factor‐like 1 gene, *LOC_Os01g15340*). The specificity in the altered gene expression profile—as evident from the over‐representation of immunity‐related genes—suggests that OsRpp30 is mainly involved in plant immunity.

We then substantiated the RNA‐seq findings with 11 selected DEGs (Figure 2h): genes encoding immunity factors (*PR5*, *PR10*, *PR1a*, *PBZ1*, *LOC_Os03g46060*), chitinases (*Cht‐3*, *CHIT8*), phenylalanine ammonia‐lyase (*PAL1*), PAMP receptor (*FLS2*), lectin‐like receptor (*LOC_Os07g03810*) and development‐related protein (*LOC_Os01g15340*). We performed RT‐qPCR using RNA extracted from leaf tissues of *OsRpp30*‐OX and WT plants with or without *P. oryzae* inoculation. Except *FLS2* and *LOC_Os07g03810*, the eight immunity and the sole development‐related (LOC*_Os01g15340*) genes were induced up to 3.5‐fold in the uninoculated *OsRpp30*‐OX compared to WT plants (Figure 2i). Interestingly, 24 hpi with *P*. *oryzae* RO1‐1, up‐regulation of all eight immunity genes was maintained or enhanced even further (*PAL1*, *PR10* and *PBZ1*) in *OsRpp30*‐OX compared to WT plants. In contrast, *FLS2* and *LOC_Os07g03810* were slightly induced, while *LOC_Os01g15340* was decreased (Figure 2i). These results suggest that protection afforded by *OsRpp30* overexpression was not because of some global alterations but likely mediated by specific (yet deciphered) mechanisms.

As an additional stringency measure, we selected 12 genes for RT‐qPCR from different functional categories (immunity, development, metabolism, cytokinesis and abiotic stress) that were *not* in our list of DEGs. None of these genes was significantly induced or suppressed in the uninoculated *OsRpp30*‐OX compared to WT plants (Figure S2). After inoculation with *P*. *oryzae* RO1‐1, only the expression of two immune‐related genes, *RbohB* and *CERK1,* was up‐regulated twofold in *OsRpp30*‐OX plants (Figure S2).

### OsRpp30 acts genetically and partially upstream of the HDT701‐mediated immunity pathway

**Figure 3:**
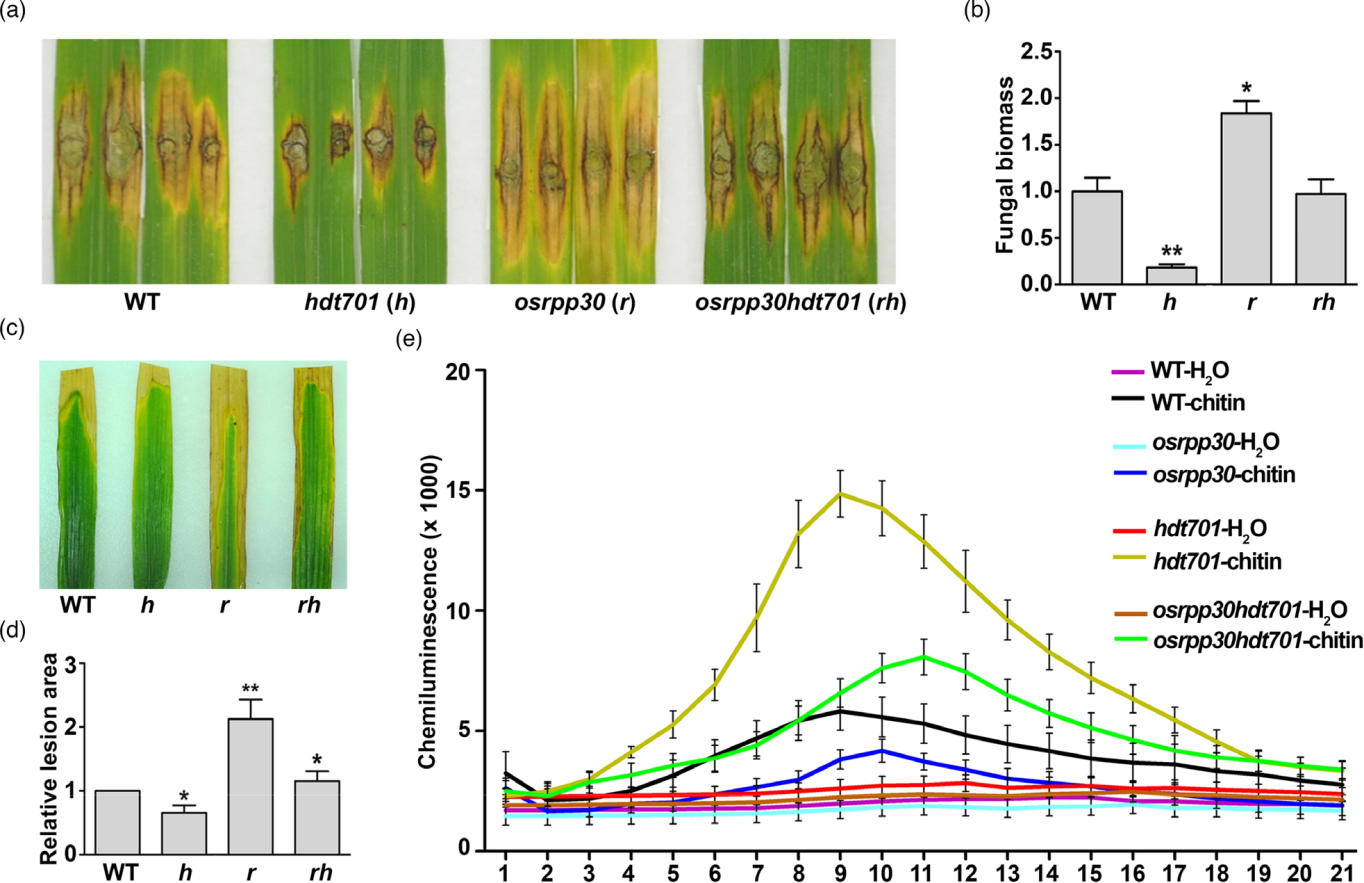
OsRpp30 is required for HDT701‐mediated plant immunity. (a) Lesion on WT, *hdt701*, *osrpp30* and *osrpp30hdt701* leaves at 13 dpi with *Pyricularia* *oryzae* RO1‐1. (b) Relative fungal biomass in the necrotic regions of the leaves in panel a. Fungal growth was assessed by qPCR of the fungal *Pot2* gene and normalized to rice *Ubiquitin* gene. The data are the mean of three replicates ± SE. Asterisks denote significant difference based on nested ANOVA (*P < 0.05, **P < 0.01). (c) Lesion on WT, *hdt701*, *osrpp30* and *osrpp30hdt701* leaves at 14 dpi with *Xoo* P6. (d) Relative lesion area of the leaves in panel c. The data are the mean of three replicates ± SE. Asterisks denote significant difference based on nested ANOVA (*P < 0.05, **P < 0.01). (e) Chitin‐induced ROS accumulation in WT, *hdt701*, *osrpp30* and *osrpp30hdt701* leaf disks. ROS was determined using the luminol‐based chemiluminescence assay with H_2_O treatment as the negative controls. Data shown are the mean of three replicates ± SE of three replicates. The experiments in panels a–e were repeated three times with similar results.

To further dissect the nexus between OsRpp30 and HDT701, we took a genetic approach and generated *osrpp30*, *hdt701* and *osrpp30hdt701* knockout mutants using the CRISPR/Cas9 strategy. We used single‐guide RNA (sgRNA) constructs to generate either an *osrpp30* or an *hdt701* single mutant or both sgRNAs in one construct to obtain the *osrpp30hdt701* double mutant (Figure S3). The T_3_‐generation mutants used in our functional assays have the following mutations: *osrpp30* with a 2‐bp deletion, *hdt701* with a 4‐bp deletion and *osrpp30hdt701* with a 1‐bp and a 7‐bp deletion, respectively (Figure S3). Because *OsRpp30* is identical to *OsRpp30‐L* and highly homologous to *OsRpp30‐S* in the sgRNA‐targeted region (nucleotides 440‐459 of *OsRpp30* coding sequence; Figure S3), we sequenced all three genes in the CRIPSR/Cas9 mutants and chose for our phenotypic analysis only those T_1_ transgenic plants that contained mutations in *OsRpp30* but neither in *OsRpp30‐L* nor *OsRpp30‐S* loci. Unlike the *Arabidopsis rpp30* mutant (Wang *et al.,* 2012), our *osrpp30* mutant did not show defects at the reproductive growth stage, likely because of multiple *OsRpp30* homologs in rice compared to the single gene copy in *Arabidopsis*. Thirteen dpi with *P*. *oryzae* RO1‐1, *hdt701* leaves showed enhanced resistance in comparison to the WT (Figure 3a), reminiscent of our finding with *HDT701* RNAi lines (Ding *et al.,* 2012); in contrast, *osrpp30* leaves were more susceptible mirroring the *OsRpp30* RNAi lines in this study (Figures 3a,b vs. 2e,g). Interestingly, *osrpp30hdt701* leaves displayed a similar level of disease resistance as the WT (Figure 3a). The *P*. *oryzae* fungal biomass in WT, *hdt701, osrpp30* and *osrpp30hdt701* were consistent with their respective resistance levels (Figure 3b).

Next, we evaluated the resistance of these mutants to *Xoo* strain P6. While *hdt701* conferred enhanced resistance to P6, *osrpp30* was susceptible to this isolate (Figure 3c). However, *osrpp30hdt701* was more resistant than *osrpp30* but slightly more susceptible than the WT (Figure 3c). The lesion areas correlated with the qualitative trends (Figure 3d). Furthermore, we analysed the accumulation of ROS in these mutants post‐PAMP elicitor chitin treatment. Leaf disks from 6‐ to 7‐week‐old rice plants were immersed in a chitin solution, and ROS accumulation was measured using the luminol chemiluminescence assay (Schwacke and Hager, 1992). ROS level was highest in *hdt701* and lowest in *osrpp30*, with the level in *osrpp30hdt701* between that in WT and *hdt701* (Figure 3e).

Our data demonstrate that OsRpp30 expression is linked to resistance towards both fungal and bacterial pathogens of rice. The phenotypic profiles of *hdt701*, *osrpp30* and *osrpp30hdt701* suggest a larger network at play and that OsRpp30 acts genetically and partially upstream of the HDT701‐mediated PAMP‐triggered immunity pathway in rice.

### HDT701 overexpression coincides with decreased acetylation level of OsRpp30

The physical interaction between HDT701 and OsRpp30 prompted us to examine whether OsRpp30 is deacetylated by HDT701. First, because of the simplicity of performing overexpression studies in bacteria and the existence of HDACs in prokaryote including *E. coli* (Jiang *et al.,* 2017; Zhao *et al.,* 2004), we inquired whether OsRpp30 is a substrate for deacetylation in *E. coli*. Accordingly, we examined the acetylation status of GST‐OsRpp30 and His‐OsRpp30 in *Escherichia coli* grown with and without trichostatin A (TSA), an HDAC inhibitor (Figure S4). Despite the non‐native context, we were encouraged to find that GST‐OsRpp30 and His‐OsRpp30 were recognized by a pan acetylation monoclonal antibody and that the level of acetylation was enhanced upon treatment with TSA (Figures S4a,b). These results indicate that OsRpp30 is subject to acetylation and deacetylation even in *E. coli*. Second, we then assessed the acetylation level of OsRpp30‐Myc in WT rice protoplasts transfected with the construct *OsRpp30‐Myc* alone or together with *HDT701‐Flag*, both driven by the 35S promoter. After pulling down OsRpp30‐Myc with anti‐Myc beads, Western blot analysis showed that the acetylation level of OsRpp30‐Myc was twofold lower in the presence of HDT701‐Flag (Figure S4c, lane 2). To substantiate this finding, we performed the same acetylation assays but with protoplasts isolated from rice plants that overexpress *HDT701* under the control of the maize *Ubiquitin* promoter and transfected with the *OsRpp30‐Myc* construct above. Again, we observed a twofold decrease in acetylation of OsRpp30‐Myc (Figure S4c, lane 3). While these experiments are preliminary, together with the finding of HDT701 interacting with OsRpp30, these results from experiments in two different contexts suggest that HDT701 could deacetylate OsRpp30 in rice.

### OsRpp30 is associated with partially purified rice RNase P

All archaeal RNase P protein subunits share homologs in eukaryotic RNase P, and Rpp30 is one of those that are present in both (Evans *et al.,* 2006; Gopalan *et al.,* 2018; Jarrous, 2002; Lai *et al.,* 2010). As the N‐terminal half of OsRpp30 shares similarity with archaeal and eukaryotic homologs, we investigated the possibility that OsRpp30 is part of the RNase P RNP.

**Figure 4:**
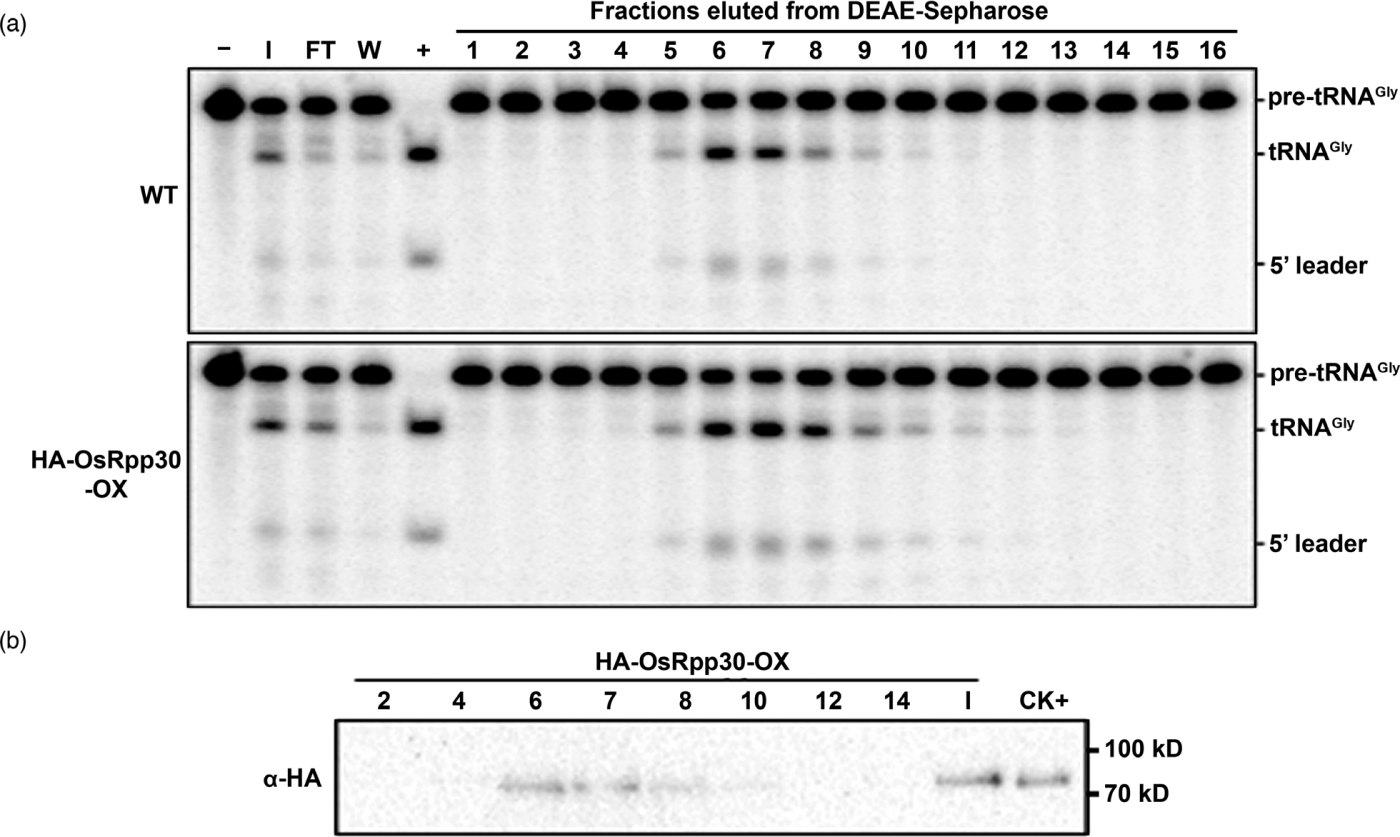
Co‐elution of HA‐OsRpp30 with rice RNase P activity. (a) Use of DEAE‐Sepharose to partially purify RNase P activity from WT and HA‐OsRpp30‐OX rice leaves. Each fraction was assayed for cleavage of pre‐tRNA^Gly^. –: negative control, pre‐tRNA^Gly^ incubated without RNase P; +: positive control, pre‐tRNA^Gly^ incubated with *in vitro* reconstituted *Escherichia* *coli* RNase P; I, FT and W: input, flow‐through and wash, respectively. (b) Western blot analysis of HA‐OsRpp30 in the indicated fractions of the HA‐OsRpp30‐OX preparation using an HA antibody (TransGen, #HT301). CK+: OsRpp30‐HA expressed in rice protoplasts and used here as a positive control.

We prepared total extracts from WT and HA‐OsRpp30‐OX rice plants and subjected each to DEAE‐Sepharose chromatography. When the eluent fractions were assayed for their ability to cleave a pre‐tRNA^Gly^, an identical strong peak of tRNA 5′‐processing activity was observed in both preparations (Figure 4a, fractions 6 to 8). The sizes of the products (Figure 4a, tRNA^Gly^ and 5' leader) generated by rice RNase P matched those of the *E. coli* RNase P RNP, which was used as a positive control (Figure 4a, lane +). Strikingly, immunoblot analysis with an HA antibody detected HA‐OsRpp30 mainly in fractions 6 to 8, which exhibited the highest RNase P activity among all fractions tested (Figure 4b).

### OsRpp30 is present in a number of wild rice species and other cereals

**Figure 5:**
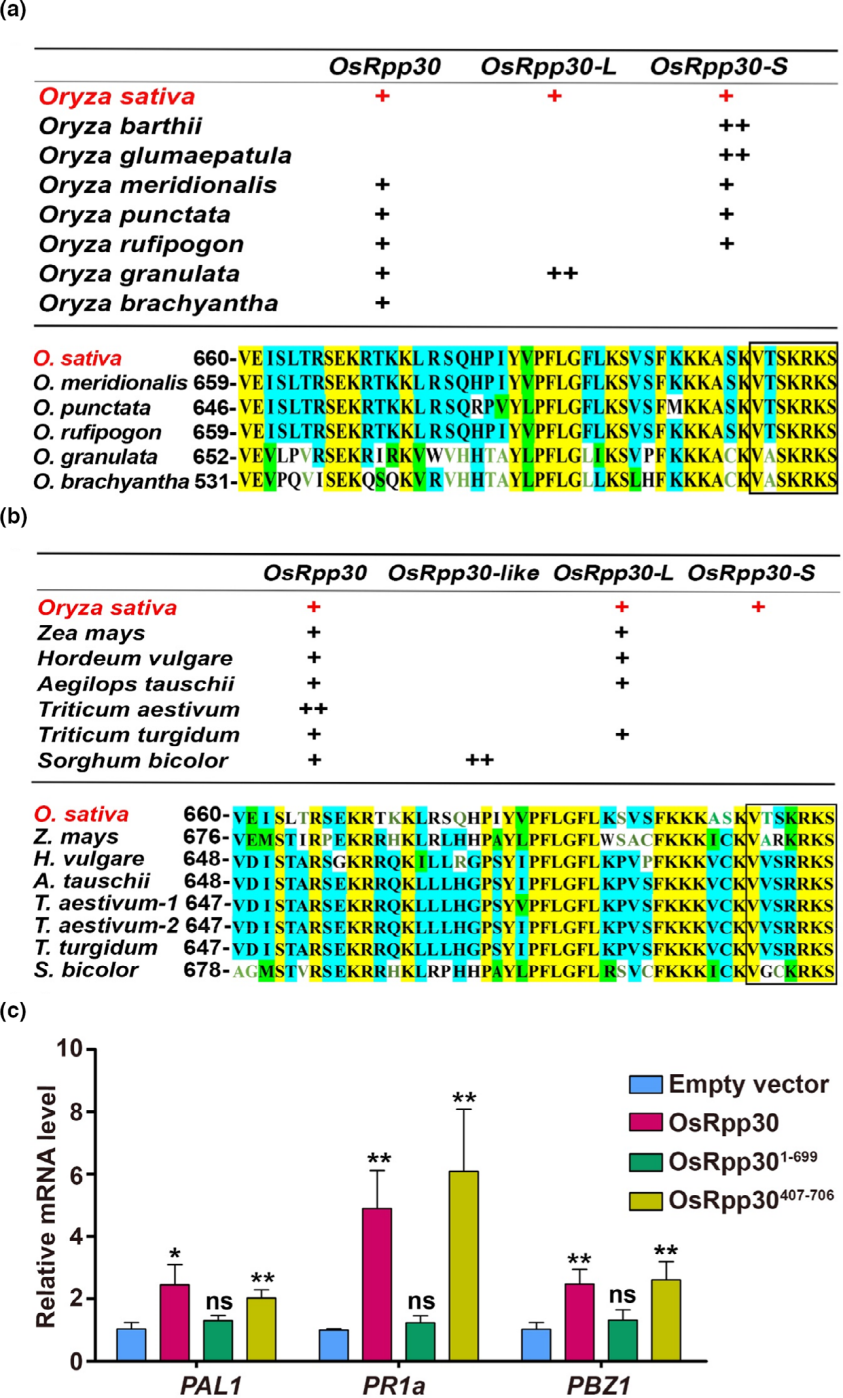
Presence of OsRpp30 in different rice species and other cereals, and its last seven aa are required for inducing defence ‐related genes expression. (a) *OsRpp30* is present in the five wild rice varieties with near‐complete sequenced genomes. Each + represents one ortholog copy. Alignment of only OsRpp30 orthologs is shown. The seven‐aa tail is boxed. (b) *OsRpp30* is present in other cereals. Each + represents one ortholog copy. Alignment of only OsRpp30 orthologs is shown. Despite low similarity elsewhere, high sequence conservation is found in the boxed seven‐aa tail of OsRpp30 from these Poaceae members. (c) The last seven aa of OsRpp30 are critical for OsRpp30‐mediated expression of defence genes. Rice protoplasts were transfected separately with an empty plasmid or a plasmid harbouring full‐length OsRpp30, OsRpp30^1‐699^ or OsRpp30^407‐706^. After a 16‐h incubation at room temperature, RNA was extracted and used for RT‐qPCR. The data are the mean of three replicates ± SE. Asterisks denote statistically significant differences based on nested ANOVA (*P < 0.05, **P < 0.01), and *ns* means not significant.

When we searched the database of wild rice genomes for *OsRpp30*, we found one copy of *OsRpp30* in five of the seven well‐annotated wild rice species, two copies of *OsRpp30‐L* in one species and one to two copies of *OsRpp30‐S* in five species (Figure 5a, table). All the *OsRpp30* orthologs in the five wild rice species share significant overall homology with *OsRpp30*, and there is almost complete conservation in the last seven aa (Figure 5a, alignment), which is essential for the interaction of OsRpp30 with HDT701 (Figures 1c,d). Moreover, in six Poaceae (grass family) members, *OsRpp30* is present in all, *OsRpp30‐L* in four and *OsRpp30‐S* in none (Figure 5b, table). In addition, *Sorghum bicolor* has two copies of a gene that we termed *OsRpp30‐like* because they code for an OsRpp30 homolog of the same length as OsRpp30 but lacking the 7‐aa tail sequence (not shown). Remarkably, the OsRpp30 orthologs in *Z. mays* (maize), *H. vulgare* (barley), *A. tauschii* (aegilops), *T. aestivum* (wheat) and *S. bicolor* (sorghum) exhibit high conservation in their last seven aa with those present in OsRpp30, despite a low overall sequence homology (Figure 5b, alignment). To determine the biological function of OsRpp30 C‐terminal half and its last seven aa, we overexpressed in rice protoplasts the full‐length OsRpp30, the truncated OsRpp30^1‐699^ lacking the last seven aa and the truncated OsRpp30^407‐706^ containing only the C‐terminus including the 7‐aa tail. Expression of full‐length OsRpp30 and OsRpp30^407‐706^, but not OsRpp30^1‐699^, led to up‐regulation of the defence genes *PAL1*, *PR1a* and *PBZ1* (Figure 5c), thus confirming a pivotal role in immunity for the last seven aa in OsRpp30.

## Discussion

We previously reported that rice HDT701 is a histone deacetylase that negatively regulates plant disease resistance (Ding *et al.,* 2012). Here, we show that HDT701 interacts with OsRpp30, a subunit of RNase P. In addition, overexpression of HDT701 in rice, either transiently or stably, coincides with decreased acetylation of OsRpp30; whether this observation is a direct or indirect action of HDT701 will require further confirmation (see below). In contrast to HDT701, OsRpp30 up‐regulates the expression of specific immunity genes and promotes resistance to *P*. *oryzae* and *Xoo*. Our overexpression and knockdown studies of HDT701 (Ding *et al.,* 2012) and OsRpp30 (this study) suggest an inverse functional relationship between these two proteins. These findings lead to the conclusion that pathogen infection induces *OsRpp30* expression, and OsRpp30 in turn promotes plant immunity by either directly or indirectly regulating expression of defence genes and ROS accumulation.

That OsRpp30 plays a role in rice immunity could be rationalized based on findings elsewhere. First, variants of the human nuclear RNase P RNP complex enhance transcription of rRNA and small ncRNA genes by interacting with RNA polymerase (RNAP) I and RNAP III, respectively (Reiner *et al.,* 2006; Reiner *et al.,* 2008; Serruya *et al.,* 2015). Therefore, it is likely that rice RNase P (either in whole or in part) containing OsRpp30 may act similarly to up‐regulate transcription of defence genes (e.g. PR1a, PR5). Second, Rpp21, Rpp29 and Pop1—three different subunits of RNase P—repress histone H3.3 chromatin assembly and therefore effect transcriptional silencing in human cells (Newhart *et al.,* 2016). Recently, Rpp29 was also shown to promote heterochromatic PTMs and repress euchromatic PTMs at specific loci (Shastrula *et al.,* 2018). There is evidence from ChIP‐seq analysis showing that Lys‐/Arg‐rich RNA‐binding proteins may function together with regulatory RNAs to remodel chromatin and control transcription (Xiao *et al.,* 2019). In light of these precedents, OsRpp30 with its 42 Lys residues could elicit transcriptional changes through epigenetic modifications, perhaps even by acting as a decoy to sequester deacetylases and therefore prolonging the euchromatin status of select loci. Lastly, mutations in Rpp30 cause sterility in *Arabidopsis* and *Drosophila* (Molla‐Herman *et al.,* 2015; Wang *et al.,* 2012). Defects in tRNA processing were not so pronounced as to warrant attributing the striking infertility phenotype to RNase P dysfunction alone. The *Drosophila* Rpp30 mutation was postulated to weaken the crosstalk between RNase P and RNAP III, thus accentuating transcription–replication conflicts, which in turn decreases expression of the piRNA genes proximal to tRNA genes (Molla‐Herman *et al.,* 2015). Absence of piRNAs—guardians of genome integrity—culminates in overexpression of repressed transposable elements, activation of DNA damage checkpoint and arrest of oogenesis. Like this ripple effect in *Drosophila*, modulation of OsRpp30 levels might alter the expression of small RNAs in rice and therefore control immunity, a testable postulate.

Other perspectives stimulated by our findings merit elaboration. First, the cultivated rice species *Oryza sativa* contains *OsRpp30‐S*, *OsRpp30‐L* and *OsRpp30*, but only expression of *OsRpp30* is up‐regulate d significantly post‐infection (Figure 2a). This finding is surprising as the promoters of *OsRpp30‐L* and *OsRpp30* are identical except for a 2‐bp difference in a 2‐kb span (no sequence conservation between these promoters and that of *OsRpp30‐S*). These near‐identical promoters may underlie the infection‐induced expression of *OsRpp30‐L* and *OsRpp30*; however, the much higher expression of *OsRpp30* suggests the existence of additional regulatory control(s) such as a remote enhancer element and/or mRNA stability. Moreover, the near‐identical sequences of *OsRpp30* and *OsRpp30‐L* render selective targeting of *OsRpp30* by RNAi difficult. However, three lines of evidence suggest that suppression of *OsRpp30* and not *OsRpp30‐L* contributes to the susceptibility phenotype of the *OsRpp30* RNAi plants. First, only *OsRpp30* is highly induced after *M. oryzae* infection (Figure 2a). Second, we found that OsRpp30‐L does not interact with HDT701 unlike OsRpp30. Lastly, *OsRpp30‐S* and *OsRpp30‐L* are intact in *osrpp30*. Thus, the identical susceptibility phenotypes of the *OsRpp30* knockout mutant and RNAi lines confirm that *OsRpp30* is the sole player (among the three homologs) in rice immunity.

Second, the evolutionary driving forces that shaped the HDT701‐OsRpp30 pairing deserve scrutiny. We recognize that HDT701 may have multiple targets and that OsRpp30 may be a substrate for additional deacetylases (e.g. HDT702) given its multiple Lys residues. The latter notion is consistent with the lack of complete deacetylation of OsRpp30 upon HDT701 overexpression.

Third, despite reports of an RNase P RNP complex in various plants, such as carrot cells, rice and wheat (Franklin *et al.,* 1995; Krehan *et al.,* 2012; Pulukkunat, 2002), only an RNA‐free, protein‐based form (PRORP) has been characterized in plants (Lechner *et al.,* 2015). However, the lack of lethality upon knocking out the single nuclear PRORP gene in the moss *Physcomitrella patens* suggests that a yet unidentified RNP form might function in the nucleus (Sugita *et al.,* 2014). This conjecture is supported by the presence of different RNase P protein subunits (e.g. Pop1, Pop5, Rpp30, Rpp29) in plant genomes. As there are only reports of a plant RNase MRP RNA (Kiss *et al.,* 1992; Krehan *et al.,* 2012), these proteins were believed to be parts of RNase MRP, the sister enzyme to RNase P that shares several protein subunits with RNase P (Lai *et al.,* 2020). Affinity tagging of OsRpp30, which co‐elutes with a tRNA‐processing activity (Figure 4), could now be exploited as a handle to obtain the elusive plant RNase P RNP.

Last, while there is overwhelming evidence for the HDT701‐OsRpp30 interaction, our efforts to identify specific acetylation sites in OsRpp30 that are acted upon by HDT701 *in vivo* have been hampered by poor yield of purified protein. We are examining newer strategies for isolating rice OsRpp30 in abundant quantities for proteomic studies.

A logical follow‐up to this study is to identify the rice acetyltransferase that acetylates OsRpp30 and the sites of acetylation/deacetylation in OsRpp30. An equally important priority is to delineate the effect of acetylation and other PTMs (e.g. succinylation; (He *et al.,* 2016a; Wang *et al.,* 2019) on OsRpp30 function in rice immunity and on RNase P activity. These experiments will help determine if pathogen‐sensing modulation of OsRpp30 function by altering the acetylation (or succinylation) status is part of a host stress response. On the other hand, it is likely that pathogens have evolved countermeasures. A recent phospho‐proteomic analysis revealed that *Xoo* infection of rice‐triggered dephosphorylation and activation of HDT701 (Hou *et al.,* 2015). Clearly, a suite of PTMs in response to infection dictates the yin‐yang balance in terms of resistance and susceptibility; the crop protection afforded by overexpression of *OsRpp30*, however, suggests that the negative role of HDT701 can be overcome to favour immunity.

Wild and cultivated rice species exhibit both distinct and shared response mechanisms to phytopathogens. Here, we found that the last seven aa are highly conserved in OsRpp30 orthologs in both wild and cultivated rice species as well as in some other cereals of agronomic importance. Somehow co‐evolution with pathogens led to a positive selection for this C‐terminal tail in a number of Poaceae species, with important payoffs for immunity to pathogens. Dissecting the molecular underpinnings of *OsRpp30* function will be key for fine‐tuning this innate immunity response. In conclusion, because *OsRpp30* overexpression in rice led to enhanced resistance to two key pathogens and the transgenic plants do not show obvious growth defects in the greenhouse and field, this study offers a new direction to generate broad‐spectrum disease‐resistant cereal crops without yield penalties to feed the growing human population.

## Methods

### Plant growth conditions

Rice (*Oryza sativa* Nipponbare) seeds were sterilized by treatment with 75% (v/v) ethanol for 1 min followed by immersion in 2% (w/v) NaCl for 40 min. After washing the seeds with sterile water, they were germinated on 1/2 Murashige and Skoog (MS) medium for 8–9 days at 26 °C with a 12‐h/12‐h light/dark photoperiod. Subsequently, the seedlings were transferred to soil and grown in a growth chamber at 26 °C and 80% relative humidity with a 12‐h/12‐h light/dark photoperiod.

### Yeast two‐hybrid assays

The Alkali‐cation^TM^ yeast transformation kit (MP Biomedicals) was used in screening for HDT701‐interacting proteins and for validating protein–protein interactions. The full‐length HDT701 cDNA was cloned into the bait vector pDBLeu, and the construct was used to transform the yeast strain Mav203. An *O. sativa* Nipponbare seedling cDNA library or the candidate interactor cDNAs were cloned into the prey vector pPC86 as described previously (Park *et al.,* 2012), and the resulting clones were used to transform the yeast strain carrying pDBLeu‐HDT701. Putative HDT701‐interacting clones grown on SD/‐Leu‐Trp medium were confirmed by growing on SD/‐Leu‐Trp‐His medium containing 20 mm 3‐amino‐1,2,4‐triazole (3‐AT), followed by DNA sequencing and bioinformatic analysis.

### Luciferase complementation imaging (LCI) analysis

LCI assay was conducted as described previously with some modifications (Chen *et al.,* 2008; Lai *et al.,* 2010). The *Agrobacterium* strain GV3101, transformed individually with the different constructs, was grown at 28° C for ˜18 h with shaking. Subsequently, the cells were harvested by centrifugation at 2500 g for 10 min, then resuspended and incubated in 10 mm MES (pH 5.7) containing 0.2 mm acetosyringone for 3 h at 25–28 °C. The cells were centrifuged again at 5000 g for 10 min and resuspended in the MES solution to yield OD_600_ ˜1. The bacterial suspension was infiltrated into *N*. *benthamiana* leaves. The RNAi suppressor P19 construct was included in the infiltration (Lakatos *et al.,* 2004) to dampen the RNA silencing response. Three days later, the treated leaves were sprayed with 5 mm luciferin for fluorescence detection, and imaging was performed using the Universal Hood II Imager (Bio‐Rad, Hercules, CA).

### Rice protoplast preparation and transfection

Rice protoplasts were isolated as previously described (He *et al.,* 2016b). Briefly, 2‐week‐old etiolated seedlings grown on 1/2 MS medium in the dark at 23° in an incubator were cut into 0.5 mm strips and incubated in a solution containing 1.0% (w/v) Cellulase R10 (Yakult, #C8260) and 0.5% (w/v) Macerozyme R10 (Yakult, #MX7351) for 4–6 h in the dark at room temperature with gentle shaking. These strips were then washed with W5 solution for 1 h at room temperature and filtered through a Miracloth layer. Protoplasts were collected by centrifugation at 1000 g for 5 min, washed once with W5 solution and suspended in MMG solution at a concentration of 1.5–2.5 × 10^6^ cells/mL. For each transfection reaction, 10 μg plasmid was added to 100 μL protoplasts, before adding 100 μL 40% (w/v) PEG + 10% (w/v) CaCl_2_ solution and incubating at room temperature for 20 min. The transfected protoplasts were washed once with W5 solution and kept in the dark for 16–24 h at room temperature before analysis.

### Confocal fluorescence microscopy

The Leica TCS‐SP5 confocal laser scanning microscope (Leica Microsystems, Heidelberg, Germany) was used to analyse the subcellular localization of HDT701‐RFP and OsRpp30‐GFP. RFP was excited at 584 nm (emission window, 589–620 nm) and GFP at 488 nm (emission window, 498–527 nm). An acousto‐optical tunable filter (AOTF) was used to control the intensity of the visible excitation laser light, and an acousto‐optical beam splitter (AOBS) was used to split the excitation light and emission light instead of filter blocks. Images were captured digitally at gain value 700 and laser intensity 4% using the Leica TCS HCX PL APO 40x/1.25 NA water‐immersion objective.

### Co‐immunoprecipitation (co‐IP)

*pYBA‐1143–OsRpp30‐HA* and *pRTV–HDT701‐Myc* or *pRTV* (empty vector) plasmids were transfected into WT rice protoplasts. At 24 h post‐transfection, total protein was extracted and subjected to co‐IP, SDS‐PAGE and immunoblotting. The HA antibody (TransGen, Beijing, China, #HT301) was used for both co‐IP and immunoblotting, and the Myc antibody (TransGen, #HT101) was used for co‐IP.

### Pathogen inoculation and disease quantification

For *P*. *oryzae* RO1‐1 spray inoculation, 3‐week‐old rice seedlings were sprayed with spore suspensions (1.5–2.0 × 10^5^ spores/mL) as described previously (Qu *et al.,* 2006). These infected leaf samples were then collected at 0, 24, 48, 72 and 96 hpi for RNA extraction and RT‐qPCR analyses. For *P*. *oryzae* RO1‐1 punch inoculation, six‐week‐old rice plants were inoculated with 10 μL of spore suspension (5 × 10^5^ spores/mL) as previously described (Park *et al.,* 2012). Fungal biomass of *P*. *oryzae* in rice leaves was assessed by qPCR of the *P*. *oryzae Pot2* gene. The *Xoo* P6 (PXO99) inoculation followed the method described elsewhere (Zhang *et al.,* 2013), and the concentration of *Xoo* P6 was 2 × 10^8^ colony forming units/mL. At 12–15 dpi when the lesions were obvious and stable, their areas were measured using ImageJ, and the average of three largest lesions was determined.

### RNA‐seq analysis

The leaves of 3‐week‐old WT and *OsRpp30*‐OX plants were collected, flash‐frozen in liquid nitrogen and stored at −80 °C. Three biological replicates were obtained for each sample. Total RNA was extracted using the RNeasy Plant Mini Kit (Qiagen, Dusseldorf, Germany, #74904), and the six sequencing libraries were prepared using NEB Next Ultra RNA Library Prep Kit for Illumina (New England Biolabs, Beverly, Massachusetts, #E7530L) following each manufacturer’s recommendations. The libraries were sequenced on an Illumina platform and paired‐end reads were generated. Raw FASTQ reads were first processed through in‐house perl scripts, during which clean reads were obtained by removing reads with adapter, ploy‐N and low quality sequences. These clean reads were then mapped to the reference rice genome (IRGSP‐1.0; http://plants.ensembl.org/index.html) using HISAT2 (https://ccb.jhu.edu/software/hisat2/manual.shtml). Only reads with ≤1 mismatch were further analysed and annotated based on the reference genome. Differential expression analysis of the two sample groups (WT and *OsRpp30*‐OX plants) was performed using the DESeq R package, version 1.10.1. A false discovery rate <0.05 and gene expression change of at least twofold by DESeq were set as the threshold for obtaining the inventory of differentially expressed genes. The RNA‐seq raw data sets were submitted to NCBI SRA database with the accession number PRJNA664877.

### Quantitative RT‐PCR (RT‐qPCR)

The Bio‐Rad CFX96 Touch Real‐Time PCR Thermocycler (Bio‐Rad Laboratories, Hercules, CA) and the TransStart Green qPCR SuperMix Kit (TransGen, #AQ101) were used for RT‐qPCR analyses. Rice RNA was first transcribed into cDNA with One‐Step gDNA Removal and cDNA Synthesis SuperMix (TransGen, #AE311,), and the cDNA was then diluted to 20 ng/μL before being used as template for RT‐qPCR. Three 25‐μL replicates were carried out per sample. The relative quantification of gene expression was calculated using the $2^{‐\Delta \Delta C_{T}}$ method according to the eligible Ct (cycle threshold; 15–35) value of target genes and *Ubiquitin*, the latter used for normalization. Primers used in these reactions are listed in Table S2.

### Detection of ROS accumulation

As previously described (Park *et al.,* 2012), leaf disks were punched from 6‐ to 7‐week‐old plants and soaked overnight in sterile distilled water. For each test condition, two leaf disks were placed in a 1.5‐mL tube containing 98 μL luminol, 1 μL horseradish peroxidase (Jackson ImmunoResearch, West Grove, PA, #112‐035‐003) and 1 μL PAMP (0.8 μm hexa‐N‐acetyl‐chitohexaose) or 1 μL water (control). Luminescence was immediately monitored at 1‐min intervals for a total of 21 min with a Glomax 20/20 luminometer (Promega). Three technical replicates were performed for each sample and treatment.

### Acetylation assay

For the experiments in *E. coli*, GST‐OsRpp30 and His‐OsRpp30 expressed in *E. coli*, grown without or with 10 nm TSA, were first isolated by immunoprecipitation with glutathione (Abcam, #ab193267) and nickel (Qiagen, #30210) sepharose beads, respectively. In subsequent immunoblotting, the antibody against GST (TransGen, #HT601) or the poly‐His tag (TransGen, #HT501) was used to assess the amount of pulled down OsRpp30 fusion proteins, and a pan acetylation monoclonal antibody (Proteintech, Rosemont, IL, #66289‐1‐Ig) was used to detect OsRpp30 acetylation. For the experiment in rice protoplasts, the *OsRpp30‐Myc* plasmid alone or together with the *HDT701‐Flag* plasmid was transfected into protoplasts of the indicated genotypes. Subsequently, OsRpp30‐Myc was purified using anti‐Myc beads for use in immunoblotting. A Myc antibody (TransGen, #HT101) was used to assess OsRpp30‐Myc amount in each lane, and the acetylation antibody was used to determine OsRpp30‐Myc acetylation.

### Partial purification of rice RNase P

One gram of 4‐week‐old rice leaves was ground in liquid nitrogen and then transferred to a large glass tube and resuspended with 9 mL of extraction buffer (EB; 20 mm Tris‐HCl, 5 mm MgCl_2_, pH 8) + 50 mm NaCl (EB50). The resuspension was homogenized with a Polytron and the lysate transferred to a 30‐mL Oakridge tube and centrifuged at 21 000 g for 30 min at 4 °C. The cleared lysate was passed through a 0.45‐μm syringe filter and loaded onto an EB50‐equilibrated 1‐mL HiTrap DEAE FF column (GE Healthcare, Uppsala, Sweden). After washing the column with 5 mL EB50, the bound components were eluted with a linear NaCl gradient (50–1000 mm in EB) using the AKTA Purifier FPLC System (GE Healthcare) at a flow rate of 1 mL/min. Sixteen 0.5‐mL fractions were collected. Subsequently, 3 μL of each fraction was assayed for RNase P activity in a final volume of 10 μL containing EB, 14 mm 2‐mercaptoethanol, 50 nm tobacco chloroplast pre‐tRNA^Gly^ (a trace amount of which was internally radiolabeled). The reaction was incubated at 37 °C for 15 min, quenched with 10 μL of stop buffer (7 m urea, 1 mm EDTA, 0.05% (w/v) bromophenol blue, 0.05% (w/v) xylene cyanol, 10% phenol) and separated on an 8% (w/v) polyacrylamide gel containing 7 m urea. The resulting gel was then imaged using the Typhoon Phosphorimager (GE Healthcare).

## Author contributions

L.B.L, V.G., G.W. and W. Liu designed research; W. Li, Y.H., L.B.L., K.Z. and Z.L. performed research; W. Li, L.B.L. and H.K. analysed data; L.D., V.G., G.W. and W. Liu supervised and coordinated research; and W. Li, L.B.L., V.G., G.W. and W. Liu wrote the paper.

## Conflict of interest

The authors declare no conflicts of interest.

## Supporting information


**Figure S1** Expression of *OsRpp30* or *HDT701* in overexpressing or RNAi transgenic rice plants
**Figure S2** Expression fold change of immunity, development and other function‐related genes in *OsRpp30*‐OX plants compared to WT plants
**Figure S3** Generation of the *osrpp30*, *hdt701* and *osrpp30hdt701* mutants by the CRISPR/Cas9 technology
**Figure S4** Overexpression of *HDT701* coincides with decreased acetylation of OsRpp30

**Table S1** Differentially expressed genes (DEGs) identified in OsRpp30‐OX plants.

**Table S2** RT‐qPCR primers used in the study.
