# Supplementary material for: The rice RNase P protein subunit Rpp30 confers broad‐spectrum resistance to fungal and bacterial pathogens
Source: Plant Biotechnol J. 2021 May 17;19(10):1988–99. doi: 10.1111/pbi.13612 (PMC8486239; doi:10.1111/pbi.13612)
Supplement: Supplementary file 1 — Figure S1 Expression of OsRpp30 or HDT701 in overexpressing or RNAi transgenic rice plants Figure S2 Expression fold change of immunity, development and other function‐related genes in OsRpp30‐OX plants compared to WT plants Figure S3 Generation of the osrpp30, hdt701 and osrpp30hdt701 mutants by the CRISPR/Cas9 technology Figure S4 Overexpression of HDT701 coincides with decreased acetylation of OsRpp30 [file PBI-19-1988-s002.pptx]

## Slide 1
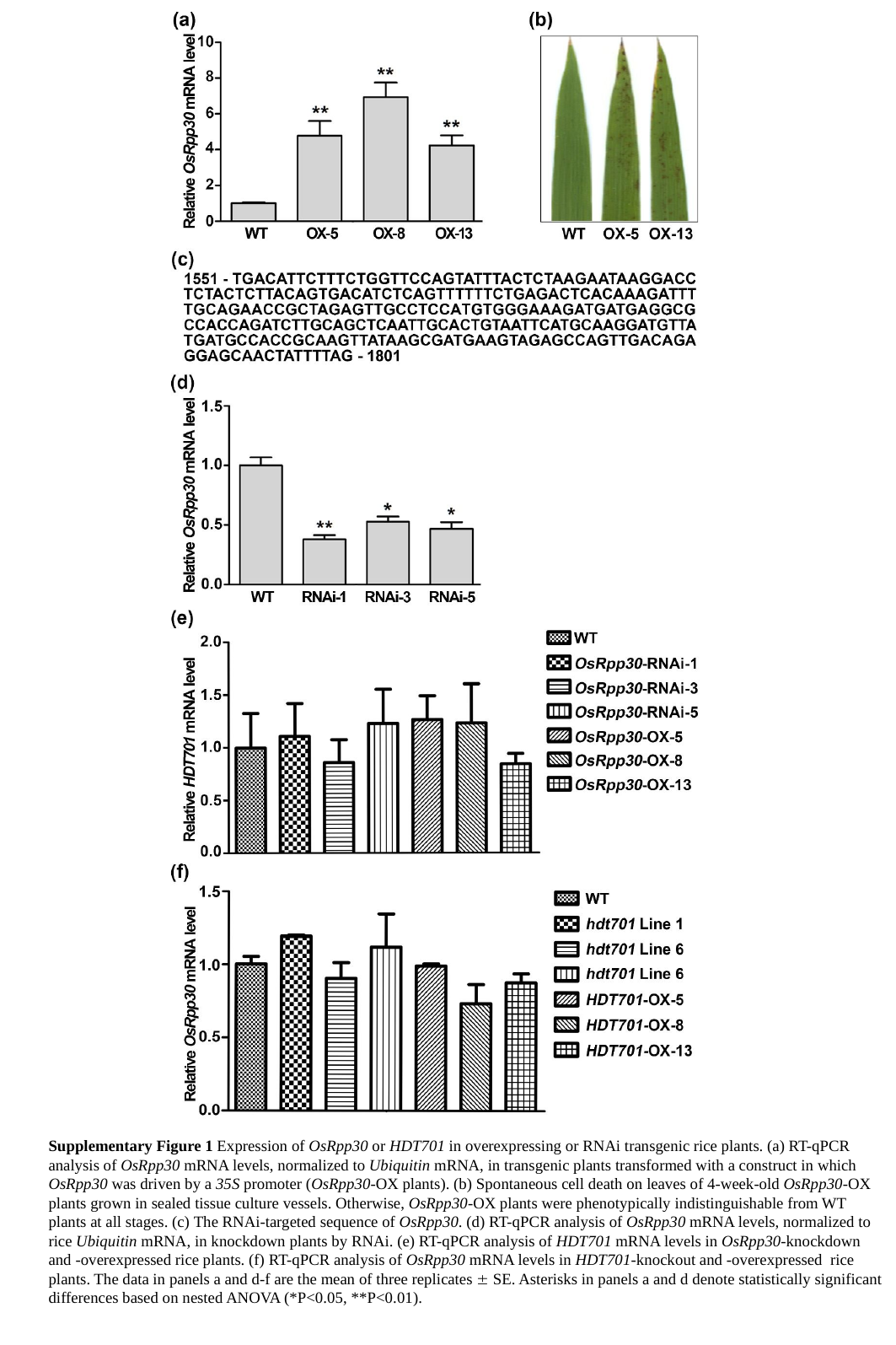

Supplementary Figure 1 Expression of OsRpp30 or HDT701 in overexpressing or RNAi transgenic rice plants. (a) RT-qPCR analysis of OsRpp30 mRNA levels, normalized to Ubiquitin mRNA, in transgenic plants transformed with a construct in which OsRpp30 was driven by a 35S promoter (OsRpp30-OX plants). (b) Spontaneous cell death on leaves of 4-week-old OsRpp30-OX plants grown in sealed tissue culture vessels. Otherwise, OsRpp30-OX plants were phenotypically indistinguishable from WT plants at all stages. (c) The RNAi-targeted sequence of OsRpp30. (d) RT-qPCR analysis of OsRpp30 mRNA levels, normalized to rice Ubiquitin mRNA, in knockdown plants by RNAi. (e) RT-qPCR analysis of HDT701 mRNA levels in OsRpp30-knockdown and -overexpressed rice plants. (f) RT-qPCR analysis of OsRpp30 mRNA levels in HDT701-knockout and -overexpressed rice plants. The data in panels a and d-f are the mean of three replicates ± SE. Asterisks in panels a and d denote statistically significant differences based on nested ANOVA (*P<0.05, **P<0.01).

## Slide 2
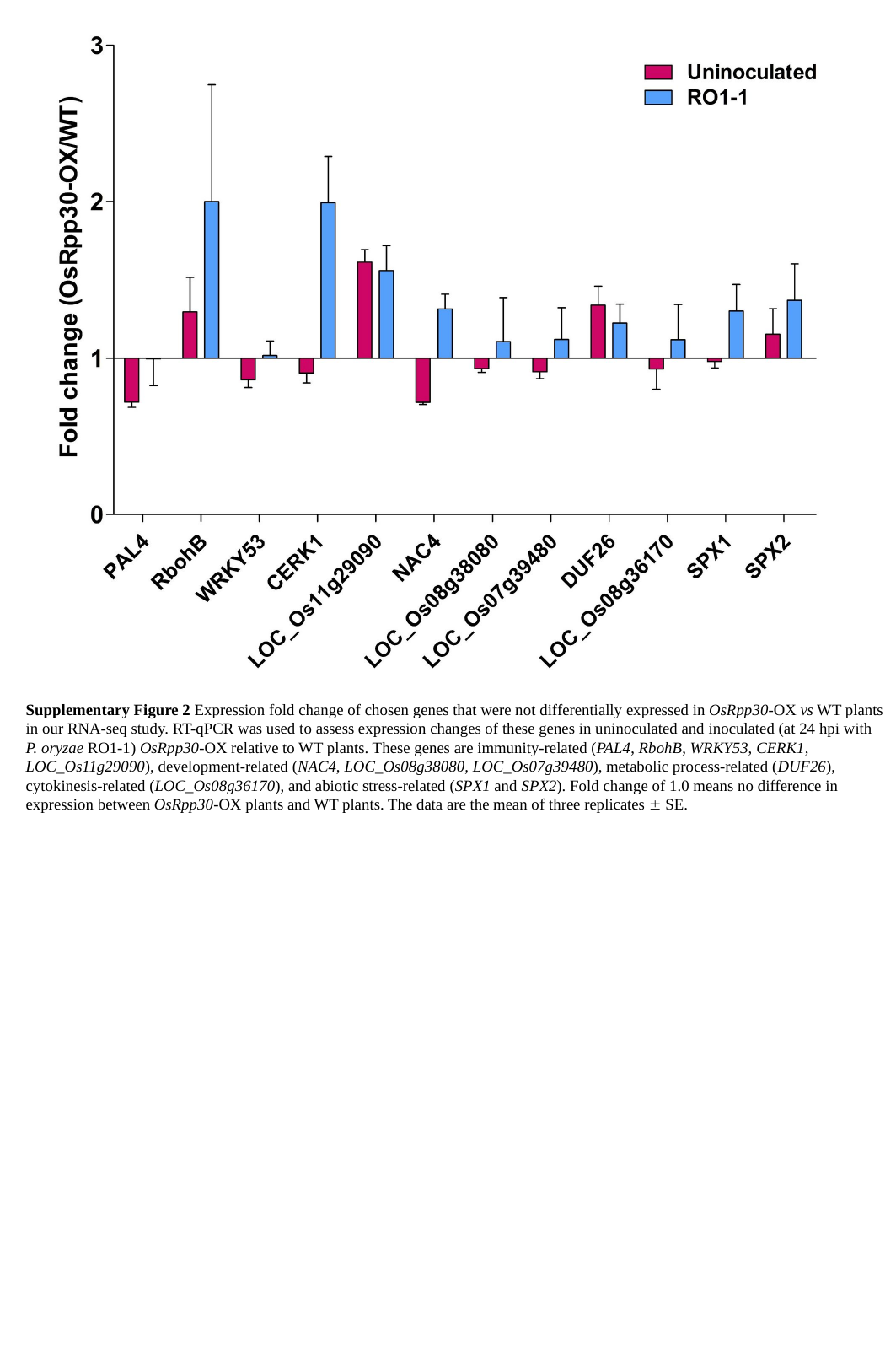

Supplementary Figure 2 Expression fold change of chosen genes that were not differentially expressed in OsRpp30-OX vs WT plants in our RNA-seq study. RT-qPCR was used to assess expression changes of these genes in uninoculated and inoculated (at 24 hpi with P. oryzae RO1-1) OsRpp30-OX relative to WT plants. These genes are immunity-related (PAL4, RbohB, WRKY53, CERK1, LOC_Os11g29090), development-related (NAC4, LOC_Os08g38080, LOC_Os07g39480), metabolic process-related (DUF26), cytokinesis-related (LOC_Os08g36170), and abiotic stress-related (SPX1 and SPX2). Fold change of 1.0 means no difference in expression between OsRpp30-OX plants and WT plants. The data are the mean of three replicates ± SE.

## Slide 3
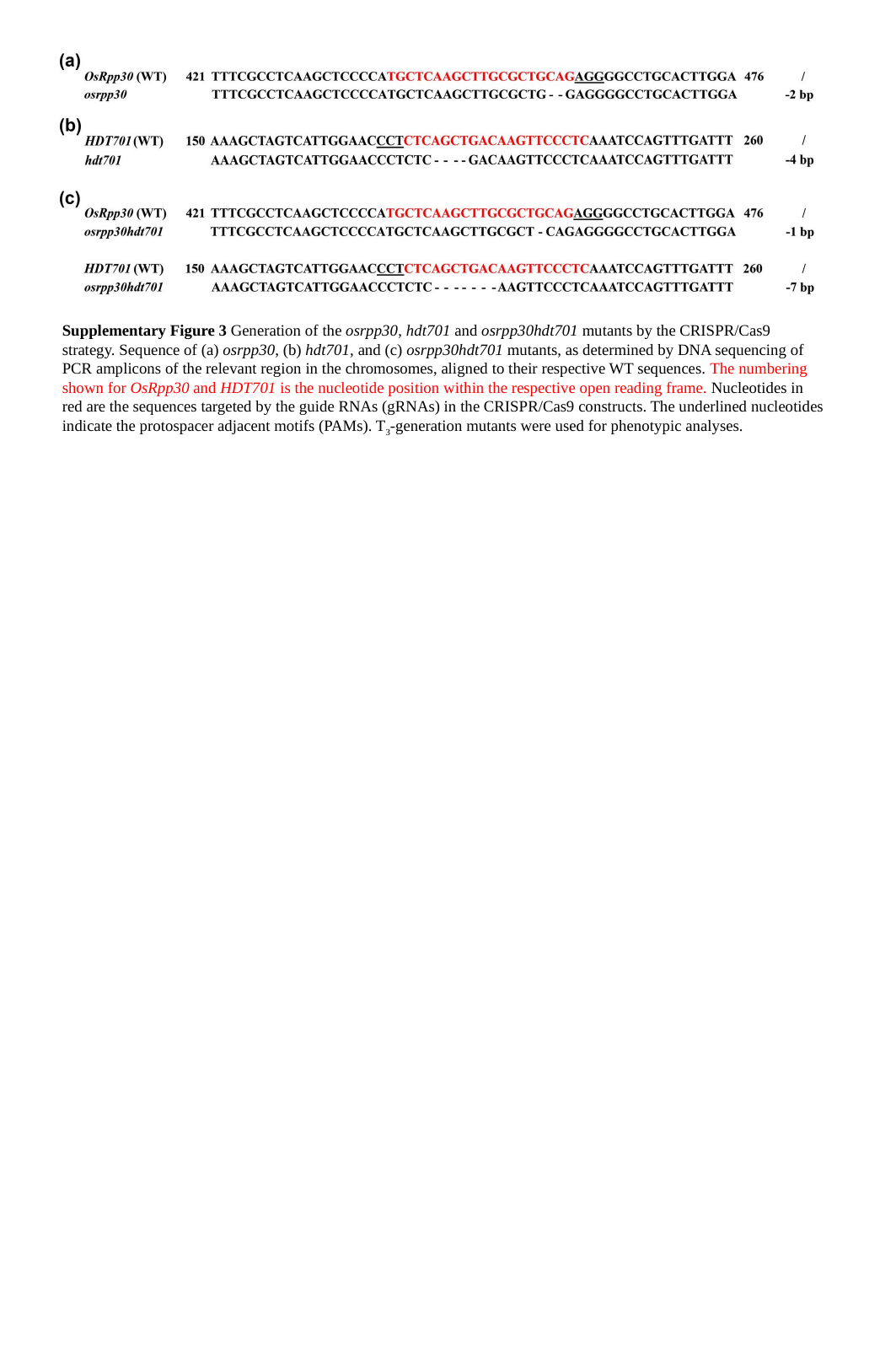

Supplementary Figure 3 Generation of the osrpp30, hdt701 and osrpp30hdt701 mutants by the CRISPR/Cas9 strategy. Sequence of (a) osrpp30, (b) hdt701, and (c) osrpp30hdt701 mutants, as determined by DNA sequencing of PCR amplicons of the relevant region in the chromosomes, aligned to their respective WT sequences. The numbering shown for OsRpp30 and HDT701 is the nucleotide position within the respective open reading frame. Nucleotides in red are the sequences targeted by the guide RNAs (gRNAs) in the CRISPR/Cas9 constructs. The underlined nucleotides indicate the protospacer adjacent motifs (PAMs). T3-generation mutants were used for phenotypic analyses.

## Slide 4
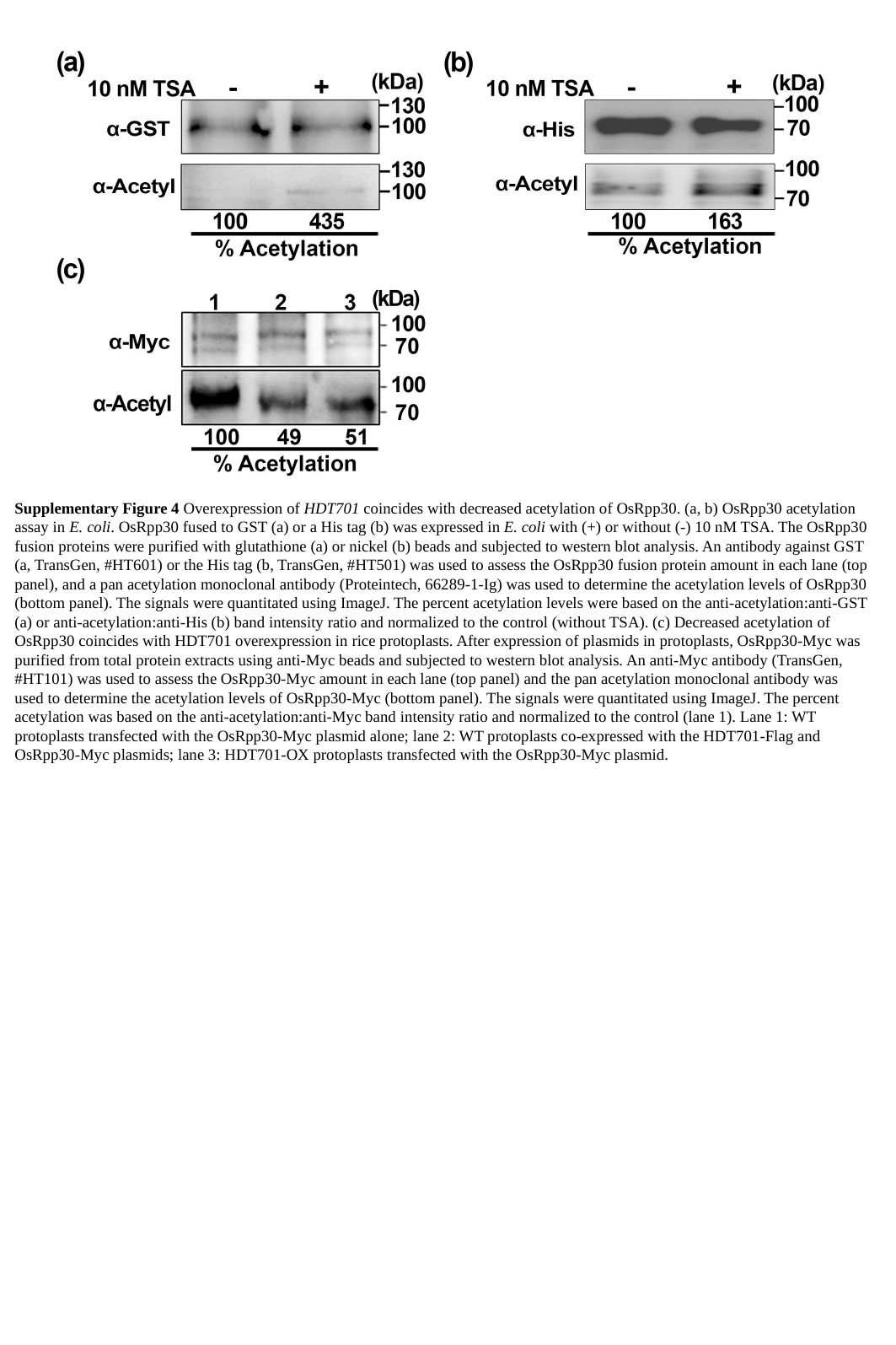

Supplementary Figure 4 Overexpression of HDT701 coincides with decreased acetylation of OsRpp30. (a, b) OsRpp30 acetylation assay in E. coli. OsRpp30 fused to GST (a) or a His tag (b) was expressed in E. coli with (+) or without (-) 10 nM TSA. The OsRpp30 fusion proteins were purified with glutathione (a) or nickel (b) beads and subjected to western blot analysis. An antibody against GST (a, TransGen, #HT601) or the His tag (b, TransGen, #HT501) was used to assess the OsRpp30 fusion protein amount in each lane (top panel), and a pan acetylation monoclonal antibody (Proteintech, 66289-1-Ig) was used to determine the acetylation levels of OsRpp30 (bottom panel). The signals were quantitated using ImageJ. The percent acetylation levels were based on the anti-acetylation:anti-GST (a) or anti-acetylation:anti-His (b) band intensity ratio and normalized to the control (without TSA). (c) Decreased acetylation of OsRpp30 coincides with HDT701 overexpression in rice protoplasts. After expression of plasmids in protoplasts, OsRpp30-Myc was purified from total protein extracts using anti-Myc beads and subjected to western blot analysis. An anti-Myc antibody (TransGen, #HT101) was used to assess the OsRpp30-Myc amount in each lane (top panel) and the pan acetylation monoclonal antibody was used to determine the acetylation levels of OsRpp30-Myc (bottom panel). The signals were quantitated using ImageJ. The percent acetylation was based on the anti-acetylation:anti-Myc band intensity ratio and normalized to the control (lane 1). Lane 1: WT protoplasts transfected with the OsRpp30-Myc plasmid alone; lane 2: WT protoplasts co-expressed with the HDT701-Flag and OsRpp30-Myc plasmids; lane 3: HDT701-OX protoplasts transfected with the OsRpp30-Myc plasmid.
